# Effect of Synthesis Method of La_1 − *x*_Sr_*x*_MnO_3_ Manganite Nanoparticles on Their Properties

**DOI:** 10.1186/s11671-017-2431-z

**Published:** 2018-01-11

**Authors:** Yulia Shlapa, Sergii Solopan, Anatolii Belous, Alexandr Tovstolytkin

**Affiliations:** 1 V. I. Vernadskii Institute of General and Inorganic Chemistry of the NAS of Ukraine, 142, Palladina ave., 32/34, Kiev, 03680 Ukraine; 2 Institute of Magnetism of the NAS of Ukraine and MES of Ukraine, 36-b Vernadsky Ave., Kiev, 03142 Ukraine

**Keywords:** Manganite nanoparticles, Sol-gel, Non-aqueous solution, Microemulsion, Magnetization, Specific loss power

## Abstract

Nanoparticles of lanthanum-strontium manganite were synthesized via different methods, namely, sol-gel method, precipitation from non-aqueous solution, and precipitation from reversal microemulsions. It was shown that the use of organic compounds and non-aqueous media allowed significantly decreasing of the crystallization temperature of nanoparticles, and the single-phased crystalline product was formed in one stage. Morphology and properties of nanoparticles depended on the method and conditions of the synthesis. The heating efficiency directly depended on the change in the magnetic parameters of nanoparticles, especially on the magnetization. Performed studies showed that each of these methods of synthesis can be used to obtain weakly agglomerated manganite nanoparticles; however, particles synthesized via sol-gel method are more promising for use as hyperthermia inducers.

**PACS:** 61.46.Df 75.75.Cd 81.20. Fw

## PACS

61.46.Df75.75.Cd81.20. Fw

## Background

Structure and properties of magnetic materials differ from those of bulk materials in the transition to the nanoscale [1]. In addition to possible practical application in various magnetic sensors, magnetic recording systems [2], magnetic nanoparticles are of particular interest in the possibilities of practical use in medicine. Researchers study many possible medical directions of their application: delivery of drugs and biological objects [3, 4], biomarkers [5], magnetic resonance imaging (MRI) [6, 7], etc.

One of the promising directions for medical application of magnetic nanoparticles is hyperthermia—locally heating the oncological tumors under the action of an alternating magnetic field to 43–45 °C, at which the tumor cells die [8]. Application of an external alternating magnetic field is accompanied by a number of problems: uneven and uncontrolled heating tumor, risk of overheating and destruction of healthy tissues, and impossibility of heating the deep-seated tumors. Therefore, in 1993, Prof. Jordan suggested the idea of magnetic hyperthermia, which consisted in the use of the magnetic nanoparticles and an alternating magnetic field [9]. In this case, the magnetic nanoparticles must be previously injected into the tumor, and such tumor must be affected by an alternating magnetic field. Particle temperature will increase by the absorption of magnetic energy and provide the local heating. However, such nanoparticles must satisfy a number of requirements: small sizes and weak agglomeration of nanoparticles; such particles must be single domain and superparamagnetic (to prevent interactions between individual nanoparticles in the absence of magnetic field), and they must effectively heat up in the alternating magnetic field to the required temperatures (43–45 °C) and demonstrate high specific loss power (SLP) values.

At present, magnetic nanoparticles of the magnetite Fe_3_O_4_ with a spinel structure are actively investigated as possible mediators of hyperthermia treatment [7, 10, 11]. Magnetite is characterized by a high value of Curie temperature (*T*_C_ ≈ 580 °C) [12]—the transition temperature from magnetic to paramagnetic state. Since magnetic nanoparticles heat up in an alternating magnetic field only when they are in a magnetic state (up to *T*_C_ point), in the case of magnetite, the heating is uncontrollable up to high temperatures. It may result in overheating and destroying the healthy tissues.

To prevent this problem, it is important to search for the alternative materials, in which the Curie point will be in the temperature range that is necessary for hyperthermia. In this case, heterosubstituted manganites of lanthanum-strontium La_1 − *x*_Sr_*x*_MnO_3_ (LSMO) with the distorted perovskite structure are of particular interest. They have the phase transition temperature near 45 °C that provides the controlled heating temperature without any additional thermoregulative devices.

Crystallization energy of materials with the perovskite structure is much higher than that for spinel structure [13]. Due to this reason, an amorphous phase is always formed in the first stage regardless of the method of synthesis of nanoparticles with the perovskite structure from solutions. Preparation of the crystalline product requires additional temperature treatment that leads to the agglomeration of the nanoparticles. Investigations described in [14] showed that formation of the crystalline structure after precipitation from aqueous solutions and further heating the powder is a multi-stage process; single-phased crystalline product is obtained at temperatures higher than 1100 °C. Such particles have large sizes and form large agglomerates. Therefore, it is relevant to search for alternative methods for synthesis of weakly agglomerated La_1 − *x*_Sr_*x*_MnO_3_ nanoparticles using non-aqueous media and organic compounds. It is possible to highlight such methods as precipitation from non-aqueous solution, microemulsion synthesis, and sol-gel method. In these cases, the formation of nanoparticles will take place either in the decomposition of previously formed organic-inorganic complexes (precipitation and sol-gel method) or in the isolated volume (microemulsions); the parameters of which can be controlled by the selection of different organic compounds.

Therefore, the aim of this study was the synthesis of nanoparticles of lanthanum-strontium manganite (La_1 − *x*_Sr_*x*_MnO_3_) via different methods (precipitation from non-aqueous solution, synthesis in microemulsion, and sol-gel method) and investigation of morphology and properties of obtained nanoparticles.

## Methods

### Methods of Synthesis

In sol-gel synthesis of LSMO manganite nanoparticles, the necessary molar amounts of metal salts La(NO_3_)_3_, Mn(NO_3_)_2_, Sr(NO_3_)_3_ were dissolved in bidistilled water. Citric acid (CA) and ethylene glycol (EG) were added to the resulting solution as gel-forming agents in a molar ratio CA/EG = 1:4. The molar ratio of salts to the gel-forming mixture was 1:10. The obtained mixture was heated at 80 °C with stirring. A polymer gel was formed as a result of polyesterification reaction, and it was pyrolyzed at 200 °C. Precursor powder obtained as a result of pyrolysis was subjected to heat treatment at different temperatures for 2 h.

For the precipitation of LSMO manganite nanoparticles from a non-aqueous medium, the concentrated aqueous solutions of metal nitrates, La(NO_3_)_3_ (*C*_La_ = 1.2 *M*), Mn(NO_3_)_2_ (*C*_Mn_ = 1.5 *M*), and Sr(NO_3_)_3_ (*C*_Sr_ = 1.6 *M*), were used as starting reagents and sodium hydroxide as the precipitator. Diethylene glycol (DEG) was used as the reaction medium. To obtain 0.01 mol of manganite, a mixture of metal nitrates was added to 1.5 mol of DEG in the three-neck flask in the argon atmosphere and heated up to 200 °C. One hundred milliliters of previously prepared sodium hydroxide solution in DEG (*C*_NaOH_ = 0.5 M) was added dropwise to the obtained mixture with constant stirring. The resulting reaction system was heated in the oil bath to 200–220 °C with stirring for 1 h and held for 1 h at this temperature. The precursor obtained after the synthesis was mixed with the oleic acid, and this mixture was cooled to room temperature. Obtained nanoparticles were separated by centrifugation, dispersed in ethyl alcohol, and dried in the air at 30–50 °C. To obtain crystalline nanoparticles, the synthesized precursor was heat-treated at different temperatures for 2 h.

To precipitate manganite LSMO nanoparticles from reversal microemulsions, aqueous solutions of La(NO_3_)_3_ (*C*_La_ = 1.2 M), Mn(NO_3_)_2_ (*C*_Mn_ = 1.5 *M*), and Sr(NO_3_)_3_ (*C*_Sr_ = 1.6 M) were used as starting reagents and cetyltrimethylammonium bromide (CTAB) and Triton X-100 as surfactants. *n*-Butanol was used as the additional surfactant which was not involved in the formation of micelles, and cyclohexane and bidistilled water were used as the solvent and dispersed medium, respectively. Concentrated aqueous ammonia solution was used as the precipitator. At the first stage, two microemulsions (M1 and M2) were prepared. They consisted of the corresponding aqueous phase (solution of salts (M1) or precipitant solution (M2)), surfactant, *n*-butanol, and cyclohexane. Percentage of microemulsion components in the case of CTAB-based microemulsion are the following: 10.5% of surfactant, 21% of *n*-butanol, 50.5% of cyclohexane, and 18% of aqueous phase and in the case of Triton X-100-based microemulsion: 15% of surfactant, 20% of *n*-butanol, 48% of cyclohexane, and 17% of aqueous phase. M2 was added dropwise to M1 with stirring for 1 h at 70 °C. The obtained precipitate was separated by centrifugation and washed several times with isopropanol and bidistilled water. Corresponding amorphous powders were heat-treated at different temperatures for 2 h.

Synthesized nanoparticles were studied by X-ray method using diffractometer DRON-4 (CuKα radiation).

Particle morphology was investigated by transmission electron microscope (TEM) JEOL JEM-1400. Average sizes and particle size distributions were calculated as described in [15] using Image Tool 3 and OriginPro 8.5 SR1 software packages.

Magnetic measurements were performed using LDJ-9500 vibrating sample magnetometer.

To determine the heating efficiency, magnetic fluids based on synthesized nanoparticles and 0.1% aqueous agarose solution were prepared. Corresponding measurements of *T*_fluid_ vs residence time *τ* dependences were obtained using magnetic coil, which generated AC magnetic field with a frequency of 300 kHz and amplitude up to 9.5 kA/m. Specific loss power (SLP) values were calculated as described in [16] using the formula:

1 $$ \mathrm{SLP}=\frac{C_{\mathrm{fluid}}\cdot {V}_{\mathrm{s}}}{m_{\mathrm{powder}}}\cdot \frac{\mathrm{d}{T}_{\mathrm{fluid}}}{\mathrm{d}\tau } $$

where d*T*_fluid_/d*τ* is an initial slope of the temperature vs time dependence, *C*_fluid_ and *V*_s_ are the volumetric specific heat and the sample volume, respectively, and *m*_powder_ is the mass of the magnetic material in the fluid.

## Results and Discussion

Synthesis with using the non-aqueous media and organic compounds has its own features. In sol-gel synthesis, nanoparticles of La-Sr manganite are obtained after pyrolysis of the polyester between citric acid and ethylene glycol, formed during the polyesterification reaction. In the case of precipitation from DEG solution, manganite nanoparticles are obtained during decomposition of corresponding complexes formed between DEG molecules and metal ions. Detailed investigations of the synthesis process are described in [17]. Two microemulsions of oil-in-water type are used in the synthesis of nanoparticles from microemulsions. Each of these microemulsions consists of the surfactant, aqueous solution of salts or precipitator, and organic non-polar solvent. Such microemulsions allow isolating the aqueous solutions in the limited volume by the formation of micelles. Synthesis of material takes place in a limited volume, in so-called nanoreactor.

**Fig. 1:**
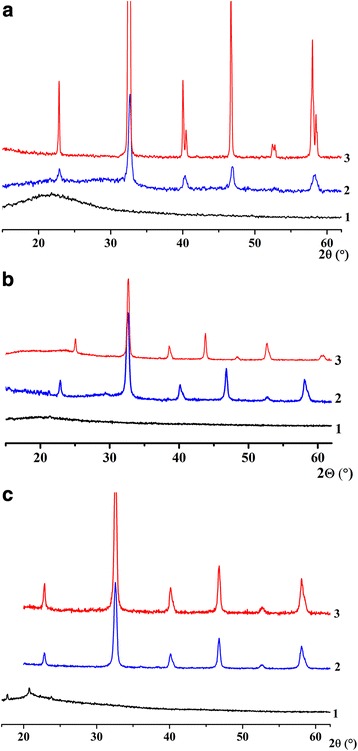
XRD data for LSMO nanoparticles, synthesized via sol-gel method (**a**), by precipitation from DEG solution (**b**), and by precipitation from reversal microemulsions (**c**): 1–200 °C, 2–600 °C, and 3–800 °C

According to XRD data, shown in Fig. 1, one can observe the formation of an amorphous non-magnetic powder after synthesis in all cases. Crystalline structure forms at a high-temperature treatment. As it can be seen from the curves (Fig. 1), the process of formation of crystalline nanoparticles is one-stage; it begins at 600 °C and finishes at 800 °C regardless of the method of synthesis. Compared to data in [14], the application of methods of synthesis from non-aqueous media makes it possible to decrease the crystallization temperature of nanoparticles and, as a consequence, to reduce their growth and agglomeration.

**Fig. 2:**
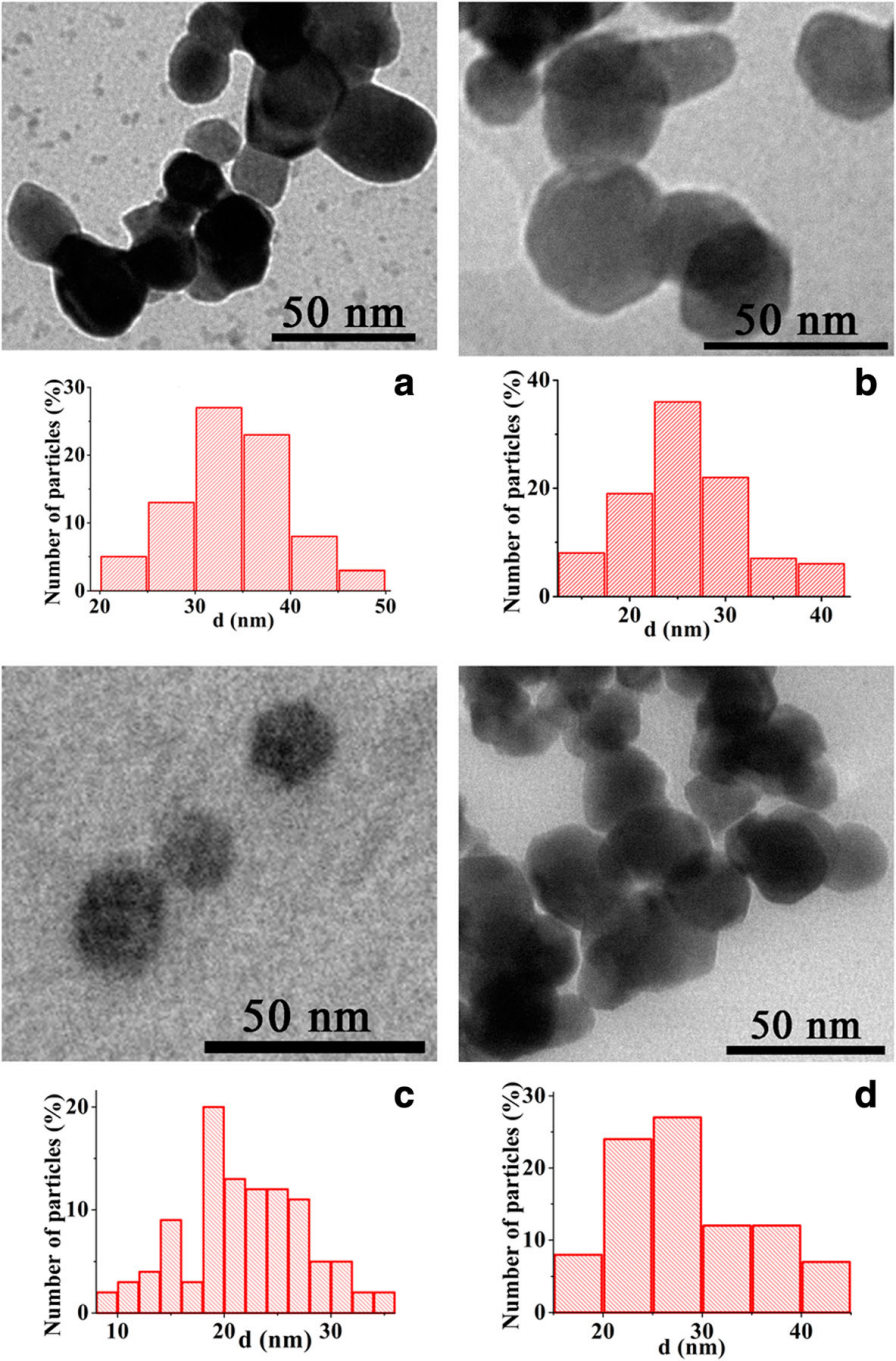
TEM images and particle size distributions of LSMO nanoparticles synthesized via sol-gel method (**a**), by precipitation from DEG solution (**b**), and by precipitation from reversal microemulsions based on Triton X-100 (**c**) and CTAB (**d**)

The results of investigation of the morphology of synthesized La_1 − *x*_Sr_*x*_MnO_3_ nanoparticles by TEM microscopy are shown in Fig. 2. The average sizes and particle size distribution are calculated, and obtained data are summarized in Table 2. TEM images shown in Fig. 2 are representative; images with large scales (100–200 nm) were used to calculate the particle size distribution.

**Fig. 3:**
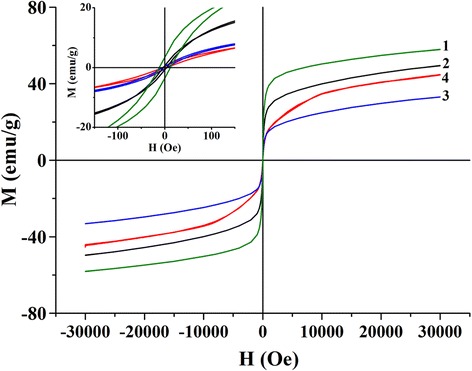
Field dependences of magnetization for LSMO nanoparticles synthesized via sol-gel method (1), by precipitation from DEG solution (2), and by precipitation from reversal microemulsions based on Triton X-100 (3) and CTAB (4). Dependences of magnetization in the weak fields are shown in the inset

As one can see from the histograms of particle size distribution (insets in Fig. 3c, d), in the case of synthesis from reversal microemulsions, the sizes of obtained nanoparticles depend on the structure of the surfactant. Triton X-100 molecules have a larger hydrophilic part compared with CTAB ones (Table 1), so they occupy a larger volume in the limited nanoreactor where the synthesis process takes place. As a result, the space available for chemical reactions becomes smaller than in traditional solution and the size of obtained product reduces.

**Table 1:** Structural formulas of surfactants for synthesis from microemulsions

Polyoxyethylenoctylphenyl ether (Triton Х-100)	
Cetyltrimethylammonium bromide (CTAB)	

Obtained results of TEM studies indicate that nanoparticles synthesized via different methods are characterized by a narrow size distribution; their average particle diameter is in the range of 20–40 nm. According to literature data, the average size of single-domain nanoparticles for manganite is about 70 nm [18]. Therefore, synthesized nanoparticles are single-domain ones, which is a necessary requirement for obtaining superparamagnetic properties.

For nanoparticles of manganite, synthesized via different methods, magnetic investigations were performed and magnetic parameters are summarized in Table 2. Field dependences of magnetization for all synthesized nanoparticles are shown in Fig. 3. As one can see from obtained results, the magnetic properties, like the morphology of particles, depend significantly on the method and conditions of synthesis. Magnetization saturation reduces with the decreasing of the particle sizes. All nanoparticles have negligible coercive force values (< 12 A/m) at the room temperature.

**Table 2:** Sizes and magnetic parameters for LSMO nanoparticles synthesized via different methods

Method of synthesis of La_1 − *x*_Sr_*x*_MnO_3_		Sol-gel synthesis	Precipitation from DEG solution	Synthesis from microemulsion based on Triton X-100	Synthesis from microemulsion based on CTAB
Average particle size		35 ± 5	25 ± 5	19 ± 5	26 ± 8
Coercive force *H*, Oe	300 K	11.8	3.9	4.2	5.2
Magnetization saturation *M*, emu/g	300 K	59	49	32	45
Blocking temperature (K)		~ 315	~ 290	~ 270	~ 290
SLP (W/g)		38	15	2	21

**Fig. 4:**
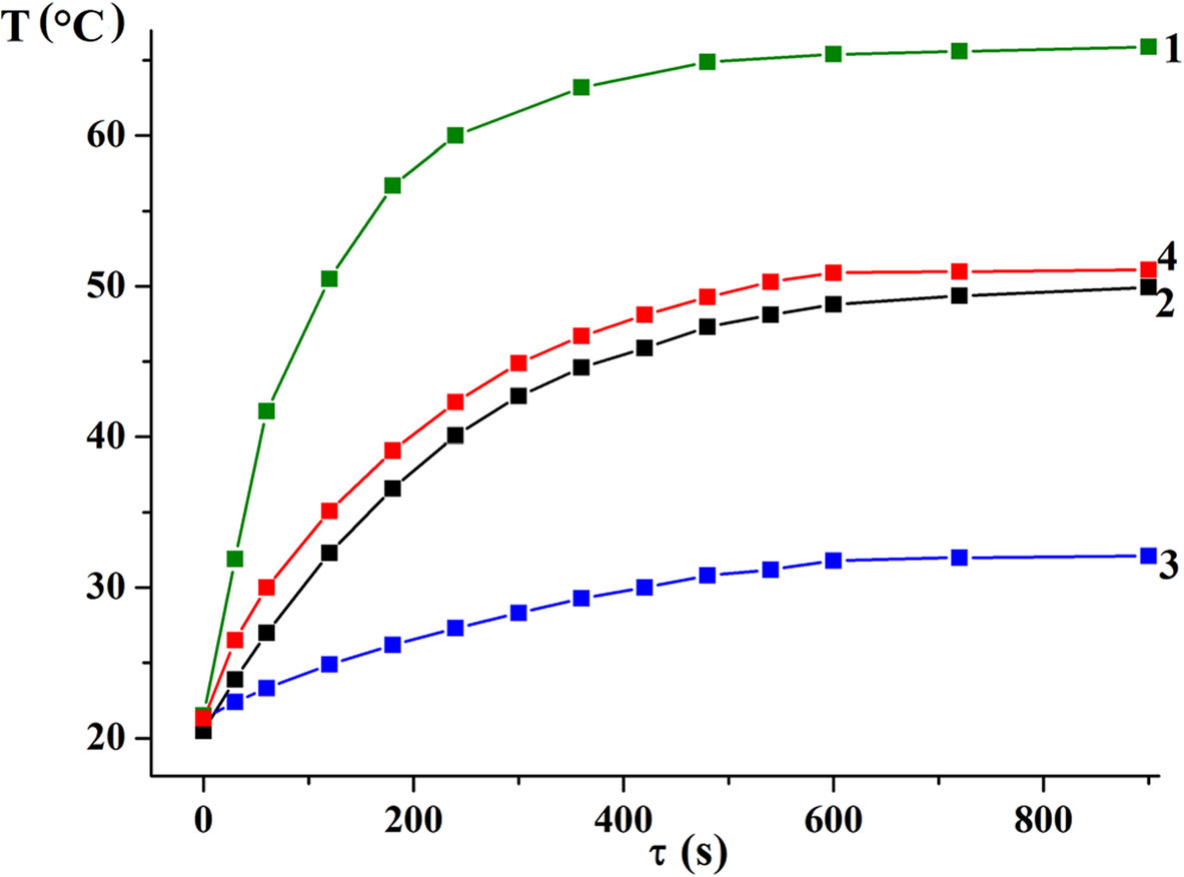
Dependences of heating temperature vs time for nanoparticles synthesized via sol-gel method (1), by precipitation from DEG solution (2), and by precipitation from reversal microemulsions based on Triton X-100 (3) and CTAB (4)

To study the heating efficiency under the action of an alternating magnetic field, magnetic fluids based on synthesized nanoparticles and agarose solution were prepared. Results of these investigations are shown in Fig. 4; calculated SLP values are summarized in Table 2. According to the obtained results, the heating efficiency depends significantly both on the magnetic properties (magnetization of nanoparticles) and on the particle morphology and size. Manganite nanoparticles synthesized via sol-gel method, which have higher magnetization values (approx. 60 emu/g) than other nanoparticles, are more efficiently heated in an alternating magnetic field (SLP value is about 38 W/g).

It is important to highlight that the heating temperature becomes stable after some time interval of action of an alternating magnetic field in all cases (Fig. 4). Maximal heating temperature depends especially on the magnetization. This is a very important result because it allows controlling the heating in the required temperature range automatically. Such approach gives a possibility to avoid the overheating and damaging of the healthy tissues in the hyperthermia treatment. However, taking into account the data of magnetic measurements, manganite nanoparticles synthesized via sol-gel method are more suitable for magnetic hyperthermia because they heat up to required temperatures (43–45 °C) in an alternating magnetic field more efficiently.

## Conclusions

LSMO manganite nanoparticles were synthesized via three methods: sol-gel, precipitation from DEG solution, and precipitation from microemulsions, where two different surfactants were used. Application of such methods allowed producing the single-phased crystalline nanoparticles in one stage at lower temperatures (up to 800 °C) compared to other methods. It was established the significant effect of method and conditions of synthesis on the morphology and properties of the nanoparticles. Calculated particle sizes are of 20–40 nm, and such particles are single-domain ones. Magnetization of nanoparticles changes in direct proportion to decreasing the particles size that affects the heating efficiency. It was shown that nanoparticles synthesized via sol-gel method better heat up in an alternating magnetic field (SLP = 38 W/g) since they have higher magnetization values. Heating temperature for all nanoparticles goes to saturation after some time that is very important for application of manganites as the hyperthermia inducers. Complex of investigations showed the possibility to synthesize weakly agglomerated, superparamagnetic manganite nanoparticles via the methods described in this paper. However, LSMO nanoparticles synthesized via the sol-gel method are more promising as the inducers in hyperthermia treatment compared with the other ones because they have better magnetic characteristics and higher heating efficiency in an alternating magnetic field (SLP = 38 W/g).

## References

[1] Ito A, Shinkai M, Honda H, Kobayashi T (2005). Medical application of functionalized magnetic nanoparticles. J Biosci Bioeng.

[2] Wu L, Jubert P-O, Berman D, Imaino W, Nelson A, Zhu H, Zhang S, Sun S (2014). Monolayer assembly of ferrimagnetic Co_*x*_Fe_3−*x*_O_4_ nanocubes for magnetic recording. Nano Lett.

[3] McBain SC, Yiu HH, Dobson J (2008). Magnetic nanoparticles for gene and drug delivery. Int J Nanomedicine.

[4] Mody VV., Cox A., Shah S., Singh A., Bevins W., Parihar H. (2014). Magnetic nanoparticle drug delivery systems for targeting tumor. Appl. Nanosci *4*:385-392./

[5] Shao H, Min C, Issadore D, Liong M, Yoon TJ, Weissleder R, Lee H (2012). Magnetic nanoparticles and microNMR for diagnostic applications. Theranostics.

[6] Na HB, Song IC, Hyeon T (2009). Inorganic nanoparticles for MRI contrast agents. Adv Mater.

[7] Blanco-Andujar C, Walter A, Cotin G, Bordeianu C, Mertz D, Felder-Flesch D, Begin-Colin S (2016). Design of iron oxide-based nanoparticles for MRI and magnetic hyperthermia. Nanomedicine.

[8] Solopan S, Belous A, Yelenich A, Bubnovskaya L, Kovelskaya A, Podoltsev A, Kondratenko I, Osinsky S (2011). Nanohyperthermia of malignant tumors. I. Lanthanum-strontium manganite magnetic fluid as potential inducer of tumor hyperthermia. Exp Oncol.

[9] Jordan A, Wust P, Fahling H, John W, Hinz A, Felix R (1993). Inductive heating of ferrimagnetic particles and magnetic fluids: physical evaluation of their potential for hyperthermia. Int J Hyperth.

[10] Hiergeist R, Andrä W, Buske N, Hergt R, Hilger I, Richter U, Kaiser W (1999). Application of magnetite ferrofluids for hyperthermia. J Magn Magn Mater.

[11] Khandhar AP, Ferguson RM, Krishnan KM (2011). Monodispersed magnetite nanoparticles optimized for magnetic fluid hyperthermia: implications in biological systems. J Appl Phys.

[12] Nikiforov VN, Koksharov YA, Polyakov SN, Malakho AP, Volkov AV, Moskvina MA, Khomutov GB, Irkhin VY (2013). Magnetism and Verwey transition in magnetite nanoparticles in thin polymer film. J Alloy Compd.

[13] Reznitskii LA, Guzei AS (1978). Thermodynamic properties of alkaline earth titanates, zirconates, and hafnates. Russ Chem Rev.

[14] Belous AG, Pashkova YV, Vunov OI, Danil'chenko KP, Yanchevskij OZ, Tovstolytkin AI, Khomenko BS, Durilin DA (2005). Effect of processing conditions on the phase transformations, structure and magnetoresistive properties of La_0.7_Sr_0.3_MnO_3±γ_ manganites. Ukrainian Chem J.

[15] Peddis D, Orrù F, Ardu A, Cannas C, Musinu A, Piccaluga G (2012). Interparticle interactions and magnetic anisotropy in cobalt ferrite nanoparticles: influence of molecular coating. Chem Mater.

[16] Veverka M, Zaveta K, Kaman O, Veverka P, Knizek K, Pollert E, Burian M, Kaspar P (2014). Magnetic heating by silica-coated Co-Zn ferrite particles. J Phys D Appl Phys.

[17] Shlapa Y, Solopan S, Yelenich O, Trachevskii V, Belous A (2016). Synthesis of ferromagnetic La_1−*x*_Sr_*x*_MnO_3_ nanoparticles by precipitation from diethylene glycol solution and their properties. J Adv Ceram.

[18] Dey P, Nath TK (2006). Tunable room temperature low-field spin polarized tunneling magnetoresistance of La_0.7_Sr_0.3_MnO_3_ nanoparticles. Appl Phys Lett.

